# Association of Patient Care with Ventilator-Associated Conditions in Critically Ill Patients: Risk Factor Analysis

**DOI:** 10.1371/journal.pone.0153060

**Published:** 2016-04-06

**Authors:** Susumu Nakahashi, Tomomi Yamada, Toru Ogura, Ken Nakajima, Kei Suzuki, Hiroshi Imai

**Affiliations:** 1 Department of Emergency and Critical Care Center, Mie University Hospital, Tsu, Mie, Japan; 2 Department of Clinical Epidemiology and Biostatistics, Graduate School of Medicine, Osaka University, Suita, Osaka, Japan; 3 Clinical Research Support Center, Mie University Hospital, Tsu, Mie, Japan; 4 Department of Radiology, Mie University School of Medicine, Tsu, Mie, Japan; University of Florida, UNITED STATES

## Abstract

**Background:**

Ventilator-associated conditions (VACs), for which new surveillance definitions and methods were issued by the Center for Disease Control and Prevention (CDC), are respiratory complications occurring in conjunction with the use of invasive mechanical ventilation and are related to adverse outcomes in critically ill patients. However, to date, risk factors for VACs have not been adequately established, leading to a need for developing a better understanding of the risks. The objective of this study was to explore care-related risk factors as a process indicator and provide valuable information pertaining to VAC preventive measures.

**Methods:**

This retrospective, single-center, cohort study was conducted in the intensive-care unit (ICU) of a university hospital in Japan. Patient data were automatically sampled using a computerized medical records system and retrospectively analyzed. Management and care-related, but not host-related, factors were exhaustively analyzed using multivariate analysis for risks of VACs. VAC correlation to mortality was also investigated.

**Results:**

Of the 3122 patients admitted in the ICU, 303 ventilated patients meeting CDC-specified eligibility criteria were included in the analysis. Thirty-seven VACs (12.2%) were found with a corresponding rate of 12.1 per 1000 ventilator days. Multivariate analysis revealed four variables related to patient care as risk factors for VACs: absence of intensivist participation in management of ventilated patients [adjusted HR (AHR): 7.325, *P* < 0.001)], using relatively higher driving pressure (AHR: 1.216, *P <* 0.001), development of edema (AHR: 2.145, *P* = 0.037), and a larger body weight increase (AHR: 0.058, *P* = 0.005). Furthermore, this research confirmed mortality differences in patients with VACs and statistically derived risks compared with those without VACs (HR: 2.623, *P* = 0.008).

**Conclusion:**

Four risk factors related to patient care were clearly identified to be the key factors for VAC preventive measures.

## Introduction

Ventilator-associated conditions (VACs), for which new surveillance definitions and methods criteria were issued by the Center for Disease Control and Prevention (CDC) in 2013 [1, 2], are “respiratory complications that occur in conjunction with use of invasive mechanical ventilation (MV)” and are associated with prolonged use of MV, prolonged intensive care unit (ICU) stay, and increased mortality [3–7]. Therefore, prevention of VACs is an important issue in managing critically ill patients. VAC surveillance, as part of VAC preventive measures, is conducted in western countries to investigate the prevalence, background, and prognosis of VACs. It is crucial to establish firm VAC preventive measures; however, to date, risk factors for VACs have not adequately been established.

For the original goal pertaining to surveillance, i.e., the achievement of medical quality improvement initiative, it is an important and pressing issue to identify risk factors for VACs. The most important characteristic of VAC diagnosis was that it included not only traditional ventilator-associated pneumonia (VAP) but also overall respiratory complications associated with the use of MV[3, 8–10]. Thus, VAC surveillance is different from traditional VAP surveillance [6, 7, 10, 11], and it is necessary to better understand the risk factors for VACs. To prevent VACs, it is particularly necessary to analyze the risk factors related to patient-care processes and management.

The objective of this study was to explore risk factors for VACs, particularly with a focus on care-related factors as a process indicator. The significance of this study is that its findings could be a basic resource for quality improvement initiatives. Therefore, it could be a valuable source to base VAC preventive measures aiming at the improvement of systems for care, treatment, and management of critically ill ventilated patients.

## Materials and Methods

**Fig 1:**
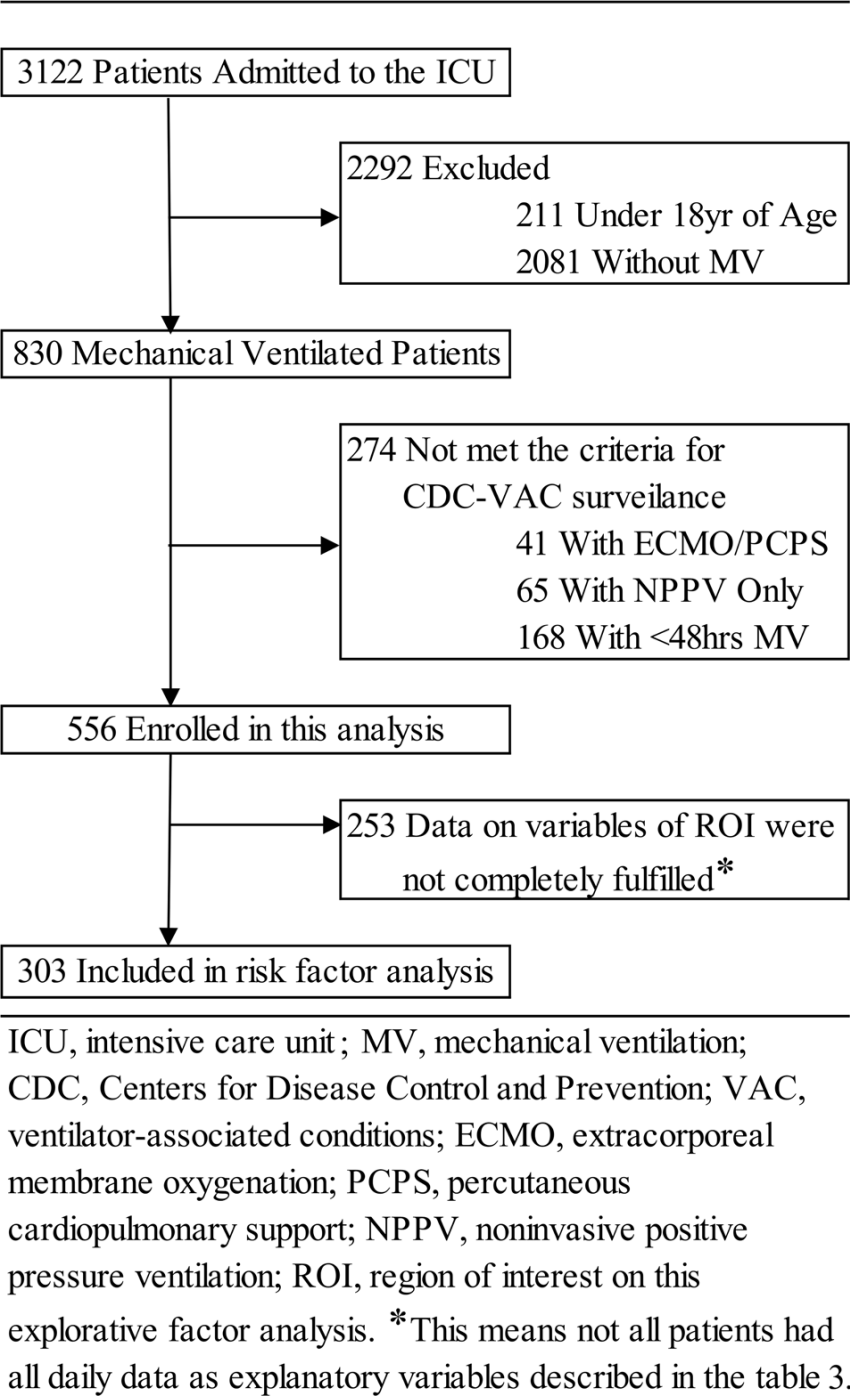
Patient disposition chart. CDC, Centers for Disease Control and Prevention; ECMO, extracorporeal membrane oxygenation; ICU, intensive-care unit; MV, mechanical ventilation; PCPS, percutaneous cardiopulmonary support; NPPV, noninvasive positive pressure ventilation; VAC, ventilator-associated conditions

This retrospective, observational, single-center study was conducted among patients who had received invasive MV between January 2012 and December 2013 in the ICU of the Mie University Hospital, an academic, urban tertiary care center located in Tsu city that has an adult multidisciplinary ICU (“semiclosed” unit) with 18 beds. The study was reviewed and approved by the Institutional Review Board of the Mie University Graduate School of Medicine that approved an opt-out consent method (IRB number 1454). This was an observational study conducted by collecting and analyzing only existing medical information and records from common and routine medical management. There was no transfer of personal information or special risk, burden, or intervention associated with participation in the study. Therefore, this study complied with the requirements for an opt-out, and informed consent was waived by the Ministry of Health, Labour and Welfare, Japan (Ministry of Education, Culture, Sports, Science and Technology, Japan: ETHICAL GUIDELINES FOR EPIDEMIOLOGICAL RESEARCH. June 17, 2002; available at https://www.niph.go.jp/wadai/ekigakurinri/guidelines.pdf. Thus this study was allowed to apply the opt-out and waiving method by the Institutional Review Board of Mie University. Accordingly, for almost all patients, the informed consent was waived (i.e., we did not obtained it). Only when the patient was in hospital during the study period (for data analysis: April 2014–December 2014), written informed consent was obtained on the advice of the Institutional Review Board. Moreover, if patients and/or the next of kin check the message board for this study in the hospital or on the website, and wish to receive any additional information, it can be made available. In such cases, we obtained verbal and written informed consent or a clarifying decision for declining testing. Selection criteria for this study were in accordance with the CDC VAC surveillance guidelines [1, 2]. Inclusion criteria included: 1) over 18 years of age and 2) receiving MV for ≥ 48 days. Exclusion criteria consisted of: 1) receiving percutaneous cardiopulmonary support (PCPS)/extracorporeal membrane oxygenation (ECMO) and 2) not receiving invasive MV but receiving non-invasive positive pressure ventilation (NPPV) (Fig 1). Patient data were automatically sampled using our computerized medical records system and then retrospectively analyzed. The data was automatically extracted from the electronic medical record system using “CLISTA” (Medical Engineering Institute Inc., Mie, Japan), a data warehouse system for medical use. This system is customized to extract and utilize medical information from the hospital’s electronic health records. In addition, the administrative databases and medical registries that record medical conditions using specific coding algorithms (billing coding), International Classification of Diseases, 10th revision (ICD-10) were also used in our study. Of the 3122 patients admitted to the ICU during the study period, 2292 were excluded because they met the exclusion criteria such as age <18 years and nonventilation (Fig 1). Furthermore, 274 patients were excluded because of conditions such as PCPS/ECMO, noninvasive positive pressure ventilation (NPPV), or ventilation of <48 h that did not meet the inclusion criteria. The remaining 556 patients were enrolled into this study (Fig 1). Finally, of the remaining 556, only patients with sufficient information of all clinical variables, discussed in detail in the next section, were included in the risk factor analysis.

### Measurements and Definition

The following text outlined the criteria for meeting the VAC definitions according to the CDC guidelines [1, 2]: if the patient has a baseline period of stability or improvement on the ventilator, defined by ≥ 2 calendar days of stable or decreased daily minimum fraction of inspired oxygen (FiO_2_) or positive end-expiratory pressure (PEEP) values. The baseline period is defined as the two calendar days immediately preceding the first day of increased daily minimum PEEP or FiO_2_. After a period of stability or improvement, as shown on the ventilator, the patient has at least one of the following indicators of worsening oxygenation: 1) an increase in the daily minimum FiO_2_ of ≥0.2 over the daily minimum FiO_2_ in the baseline period, sustained for ≥ 2 calendar days; and 2) an increase in the daily minimum PEEP values of ≥3 cmH_2_O over the daily minimum PEEP in the baseline period† sustained for ≥ 2 calendar days. Host-related, environmental, and management and care-related factors were analyzed to identify the risk factors for VACs. Comorbid disease status was measured using the International Classification of Diseases, version 10 (ICD-10), of the Charlson comorbidity index [12]. The level of activities of daily living (ADL) on admission was assessed using the Barthel index. The nurse/artificial life support devices (ALS) ratio was defined as the ratio of ALS to the number of daily staffing nurses. Hemodiafiltration, intraaortic balloon pumping (IABP), and invasive MV were included as ALS. The level of sedation during the day was assessed using the Richmond Agitation Sedation Scale (RASS). Body weight (BW) change was defined as peak minus admission BW. The rate of oral care performance was calculated as follows: total number of oral care/3 (times) × total MV days; oral care three times daily is routine practice in our ICU. Moreover, this study attempted to confirm whether VAC, with risks statistically derived from this analysis, was related to mortality as in previous studies [4–7, 10, 11]. The diagnosis that we explicitly considered were defined according to the previous research of VAC [6] as follows: 1) pneumonia was identified by imaging, cultures, initiation of antibiotics, and clinical documentation; 2) atelectasis was identified by imaging, ultrasonography, blood gas analysis, tidal volume, auscultation, bronchoscopy, and explicit clinical documentation; 3) pleural effusion was identified by imaging, ultrasonography, auscultation, percussion, and chest tube placement; and 4) ARDS (by the Berlin definition [13]) was identified by imaging, timing, ultrasonography, and oxygenation.

### Statistical Analysis

For the investigation of independent risk factors affecting the incidence of VAC, a Cox proportional hazards analysis with the exception of severity of illness (APACHE II score), which was included in all models, was used. The adjustment of the APACHE II core was necessary because the severity of illness would lead to a bias. For identification of the variables set in the model, a stepwise variable selection method was applied after forcing APACHE II score in the model. All statistical tests were two-sided, and significance was defined as *p* < 0.05. Logarithmic transformation was performed as needed to adjust normality. The period of observation was set from the initiation of MV to the onset of VAC or weaning off MV.

VAC includes traditional VAP. Risks of traditional VAP differ between early-VAP (onset of ≤4 days) and late-VAP (onset of >4 days). In this regard, the risk of VACs, including VAPs, will also differ between the early-onset VAC and other types of VAC. Therefore, risk exploration was conducted not only by methods that do not involve the classification of VACs but also by the classification of VACs according to incidence within 4 days (early incidence) and beyond 4 days (late incidence) using a polychotomous logistic regression analysis (stepwise variable selection method) with a forced entry of the APACHE score.

A cumulative survival curve was estimated using the Kaplan–Meier method and was compared using the log-rank test. Hazard ratios and 95% confidence intervals (CIs) were estimated using the Cox proportional hazards analysis, with a VAC as a time-dependent covariate. The follow-up period extended from the initiation of MV to the in-hospital death or discharge. The overall survival from a defined time point was evaluated. SAS software v9.3 for Windows (SAS Institute, Cary, North Carolina) was used for statistical analysis.

## Results

### Baseline Characteristics of Study Patients

In total, 556 patients were screened initially, of which 253 patients were excluded; data for the remaining 303 patients, shown to have sufficient information on variables in the area of interest for this analysis, were calculated using statistical analysis (Fig 1). Baseline characteristics and outcomes in patients with and without VACs are shown in Table 1. No between-group differences in age, sex, BW, and comorbidities were found. Thirty-seven VACs were identified (12.2%), with a corresponding rate of 12.1 per 1000 MV days (Table 2). VAC onset occurred, on an average at 7.1 ± 6.3 MV days. A comparison of patients with and without VACs demonstrated a significantly higher baseline Acute Physiology and Chronic Health Evaluation (APACHE) II score in those with VACs. In contrast, the baseline ADL score in patients with VACs was lower than in those without VACs. Respiratory function in patients with VACs was associated with lower oxygenation. Compared with patients without VACs, patients with VACs had a longer MV duration and ICU stay. Furthermore, 29.7% (11/37) of patients with VACs died compared to 11.7% (31/266) of patients without VACs (Table 1). Table 2 presents the etiology and distribution of the major pathological symptoms of VACs, including pneumonia, atelectasis, acute respiratory distress syndrome (ARDS), and pleural effusion. Forty percent of patients with VACs were complicated because of pleural fluid accumulation, regardless of the amount of fluid accumulated.

**Table 1:** Baseline demographics and outcome.

	No VAC	VAC^✽^	*p* Value
	n = 266	n = 37	
**Demographics**			
Age, y, mean (SD)	64.9 (14.3)	63.4 (15.3)	0.5524
Female, No. (%)	84 (31.6)	12 (32.4)	1.0000
Body weight, kg, mean (SD)	57.3 (12.2)	61.5 (11.0)	0.0438
BMI, mean (SD)	21.9 (3.7)	23.0 (4.1)	0.1056
Brinkman index, mean (SD)	447.9 (662.2)	365.9 (476.8)	0.4682
APACHE Ⅱ score, mean (SD)	16.8 (6.2)	21.8 (11.0)	< 0.0001
Charlson comorbidity index, mean (SD)	1.7 (2.3)	1.9 (2.5)	0.7122
ADL, mean (SD)^†^	47.0 (48.1)	22.4 (40.2)	0.0034
**Respiratory function**			
P/F, mean (SD)	292.0 (129.6)	217.3 (110.8)	0.0018
OI, mL/cmH_2_O, mean (SD)	2.41 (2.12)	3.44 (2.36)	0.0117
Tidal volume, mL, mean (SD)	8.5 (1.5)	8.2 (1.3)	0.2526
Cdyn, mean (SD)	43.9 (21.0)	38.6 (12.0)	0.2221
**Outcome**			
Duration of MV, d, mean (SD)	8.6 (22.7)	20.8 (16.4)	0.0017
ICU length of stay, d, mean (SD)	10.0 (18.6)	25.2 (14.2)	< 0.0001
Hospital length of stay, d, mean (SD)	36.7 (39.7)	47.0 (32.5)	0.1317
Mortality-hospital, No. (%)	31 (11.7)	11 (29.7)	0.0084

Data are presented as n (%), except where noted otherwise. SD, standard deviation; MV, mechanical ventilation; APACHE, acute physiology and chronic health evaluation; ADL, activities of daily living; BMI, body Mass Index; ICU, intensive care units; P/F, the ratio of arterial oxygen concentration to the fraction of inspired oxygen; OI, oxygen index; Cdyn, dynamic compliance of respiratory system; VAC, ventilator-associated conditions.

*Includes episodes that meet criteria for infected VAC (iVAC) and/or Possible or Probable Pneumonia: pVAP (both subtypes of VAC: iVAC is VAC with general evidence of infection and pVAP is iVAC with lab evidence of pneumonia).

†Activities of daily living (ADL) were assessed using the Barthel index.

**Table 2:** Episodes of ventilator-assocated conditions.

	VAC^✽^
Number of episodes	37 (12.2)
Incidence rate (per 1,000 MV)	12.1
Etiology	
Pneumonia	14 (37.8)
Atelectasis	8 (21.6)
Acute respiratory distress syndrome	5 (13.5)
Cardiogenic pulmonary edema	0 (0.0)
Pleural fluid	15 (40.5)

Data are presented as n (%), except where noted otherwise. VAC, ventilator-associated conditions; MV, mechanical ventilation.

* Includes episodes that meet criteria for infected VAC (iVAC) and/or Possible or Probable Pneumonia: pVAP (both subtypes of VAC: iVAC is VAC with general evidence of infection and pVAP is iVAC with lab evidence of pneumonia).

### Risk Factors for VAC

The results of univariate analysis are summarized in Table 3. Among host-related factors, body mass index (BMI), APACHE II score, and multiple trauma were statistically identified. Furthermore, six variables pertaining to patient care were identified as risk factors (Table 3), suggesting that care-related risk factors varied widely and were subject to environmental factors, MV settings, and adjunctive therapies for respiratory management. A statistically significant relationship existed between VACs and each of the following: (1) patient non-exposure to intensivists, (2) relatively deeper mean RASS level during the day, (3) vomiting, (4) development of edema, (5) a larger BW increase, and (6) a higher driving pressure (ΔP). BW changes were as follows: 3.67 ± 4.27 kg (5.9%) in patients with VACs and 1.2 ± 2.20 kg (2.1%) in patients without VACs. ΔP was also greater in patients with VACs than in those without VACs (Table 3). Oral care and semirecumbent position, which are major components of the VAP prevention program (called the VAP bundle), were not presumed to be risk factors for VAC.

**Table 3:** Ventilator-associated conditions univariate analysis.

	No VAC	VAC^✽^	HR	95%CI	*p* Value
	n = 266	n = 37			
**Demographics**					
Age, y, mean (SD)	64.9 (14.3)	63.4 (15.3)	0.997	0.975–1.019	0.768
BMI, mean (SD)	21.9 (3.7)	23 (4.1)	1.089	1.002–1.184	0.045
Brinkman index, mean (SD)					
0	123 (46.2)	19 (51.4)	1.063	0.557–2.029	0.853
1–399	39 (14.7)	4 (10.8)	0.684	0.242–1.931	0.471
≥400	104 (39.1)	14 (37.8)	1.126	0.578–2.195	0.728
APACHE Ⅱ score, mean (SD)	16.8 (6.2)	21.8 (11.0)	1.050	1.019–1.081	0.003
≥20	75 (28.2)	20 (54.1)	1.822	0.953–3.483	0.066
≥15	163 (61.3)	26 (70.3)	2.041	0.930–4.480	0.069
Charlson comorbidity index					
0	80 (30.1)	16 (43.2)	0.581	0.303–1.115	0.099
≥1	186 (69.9)	21 (56.8)	1.217	0.6372–2.324	0.551
ADL^†^					
>60	124 (46.6)	8 (21.6)	0.573	0.261–1.258	0.160
<40	137 (51.5)	28 (75.7)	1.667	0.784–3.545	0.180
**Comorbidities**					
Chronic heart failure	23 (8.6)	1 (2.7)	0.324	0.044–2.365	0.241
Chronic lung disease	24 (9.0)	1 (2.8)	0.267	0.037–1.955	0.163
Diabetes	54 (20.3)	6 (16.2)	1.000	0.417–2.401	1.000
Chronic kidney disease	32 (12.0)	2 (5.4)	0.345	0.082–1.448	0.128
Liver disease	57 (21.4)	8 (21.6)	0.747	0.336–1.660	0.473
**ICU of admission**					
Medical	50 (18.8)	13 (35.1)	1.445	0.678–2.628	0.402
Surgical (Elective)	151 (56.8)	12 (32.4)	0.606	0.304–1.210	0.152
Surgical (Emergency)	65 (24.4)	12 (32.4)	1.302	0.651–2.605	0.454
**Primary admission diagnosis**^**‡**^					
Pneumonia	14 (5.3)	2 (5.4)	0.674	0.162–2.812	0.586
Respiratory failure	19 (7.1)	8 (21.6)	1.872	0.854–4.105	0.112
Myocardial infarction/congestive heart failure	9 (3.4)	0 (0.0)	0.000	0.000–0.000	0.176
Coronary artery disease	18 (6.8)	0 (0.0)	0.000	0.000–0.000	0.067
Cerebrovascular disease	37 (13.9)	4 (10.8)	0.674	0.239–1.905	0.454
Renal failure	13 (4.9)	5 (13.5)	1.839	0.713–4.740	0.201
Multiple trauma	16 (6.0)	8 (21.6)	2.595	1.180–5.708	0.014
**Variables**					
Iintensivists participation in their care^†^	180 (67.6)	11 (29.7)	3.380	1.682–6.794	< 0.001
Nurs:ALS ratio, mean (SD)^‡^	1.53 (0.49)	1.6 (0.54)	1.139	0.513–2.528	0.749
NMBAs	50 (18.8)	8 (21.6)	1.811	0.871–3.768	0.107
Daily RASS, mean (SD)	-2.5 (1.4)	-3.4 (1.5)	0.722	0.557–0.936	0.013
Disorientation / cognitive disorder	74 (27.8)	22 (59.5)	1.652	0.847–3.219	0.137
Vomiting	20 (7.5)	0 (0.0)	0.000	0.000–0.000	0.042
Timing of tracheostomy, d, mean (SD)	5.4 (4.2)	10.5 (3.5)	1.004	0.990–1.017	0.583
Inhaltion via a nebulizer	20 (16.5)	2 (5.4)	0.449	0.108–1.873	0.259
Edema	61 (22.9)	20 (54.1)	2.250	1.168–4.336	0.013
Changes in body weigh^§^	1.2 (2.20)	3.67 (4.27)	17.841	2.443–130.296	< 0.001
≥2 kg	49 (18.4)	17 (45.9)	1.571	0.815–3.034	0.174
≥3kg	35 (13.2)	15 (40.5)	1.777	0.910–3.470	0.089
Urine output					
mL, mean (SD)	1776 (809.8)	1569 (929.9)	1.000	0.999–1.000	0.344
mL/kg/hr, mean (SD)	1.26 (0.82)	1.15 (1.00)	0.738	0.349–1.562	0.427
Driving pressure, cmH_2_O, mean (SD)	11.7 (2.8)	13.4 (3.6)	1.122	1.035–1.217	0.007
Oral care, mean (SD)	0.39 (0.99)	0.448 (0.38)	1.759	0.881–3.513	0.105
Semirecumbent position, hr, mean (SD)	3.59 (2.29)	3.46 (1.01)	1.057	0.842–1.326	0.633

Data are presented as n (%), except where noted otherwise. SD, standard deviation; CI, confidence interval; HR, hazard ratio; APACHE, acute physiology and chronic health evaluation; ADL, activities of daily living; BMI, body Mass Index; VAC, ventilator-associated conditions; ALS, artificial life support devices; NMBAs, neuromuscular blocking agents; RASS, Richmond agitation sedation scale.

* Includes episodes that meet criteria for infected VAC (iVAC) and/or Possible or Probable Pneumonia: pVAP (both subtypes of VAC: iVAC is VAC with general evidence of infection and pVAP is iVAC with lab evidence of pneumonia). †Activities of daily living (ADL) were assessed using the Barthel index.

‡Recorded as the diagnosis most representative of the reason for admission in the ICU.

†Patients experienced greater exposure to intensivists (attending physician specialists in critical care medicine).

§The ratio of artificial life support devices to the number of daily staffing nurses.

#Body weight change was defined as peak minus admission body weight.

A Cox proportional hazards analysis with the exception of the APACHE II score, which was included in all models, showed the following results: At step 0, multiple trauma, intensivist participation, RASS, edema, BW change, ΔP, peak inspiratory pressure (PIP), and vomiting showed a significant association with the endpoint adjustment for the APACHE II score. The most significant factor for BW change was first entered into the Cox model [Akaike's Information Criterion (AIC): 327.76]. For step 1, multiple trauma, intensivist participation, RASS, ΔP, PIP, and BMI were significant after adjusting for the APACHE II score and BW change. Among them, intensivist participation showed the most significant and was entered into the Cox model (AIC: 316.42). In step 2, edema, ΔP, PIP, heart failure, and timing of tracheostomy show a significant association with the endpoint adjustment for the APACHE II score, BW change, and intensivist participation. Next, the most significant factor for ΔP was entered into the Cox model (AIC: 301.37). In step 3, edema was significant after adjusting for the APACHE II score, BW change, intensivist participation, and ΔP. Edema demonstrated the highest significance and was entered into the Cox model (AIC: 299.07). In the next step, none of the factors was significantly associated with the VAC after adjusting for the APACHE II score, BW change, intensivist participation, ΔP, and edema. Thus, the stepwise analysis was terminated at step 3. Table 4 presents the results of the multivariate analysis using the Cox proportional hazard analysis. This table shows the estimates and P values when five final variables selected by the stepwise procedure (that is, statistically significant) were set to the model. Predominantly, care-related risk factors likely impacted the incidence of VACs. Risk varied with the participation of intensivists. Furthermore, results from this Cox proportional hazard analysis indicated that a relatively higher mean ΔP level, development of edema, and large BW increase were major risk factors for VACs (Table 4). Accordingly, on multivariate analyses in the polychotomous logistic regression analysis, a similar trend was indicated for the following risk factors: APACHE II score [odds ratio (OR): 1.068, *p* < 0.001], participation of intensivists (OR: 0.079, *p* < 0.001), ΔP (OR: 1.198, *p* < 0.001), edema (OR: 0.223, *p* = 0.001), and change in BW (OR: 0.071, *p* = 0.004) was associated with increased risk of VACs.

**Table 4:** Risk of ventilator-associated conditions: VAC using Cox proportional hazard model (Stepwise Variable Selection) (n = 303).

Risk factor	Adujusted	(95% CI)	*P* Value
	HR		
APACHE Ⅱ score	1.063	1.021–1.106	0.010
Insufficient participation of Intensivists in their care	7.325	3.264–16.440	< .0001
Higher driving pressure	1.216	1.109–1.333	< .0001
Changes in body weight increases	0.058	0.008–0.430	0.005
Development of edema	2.145	1.045–4.401	0.037

HR, hazard ratio; CI, confidence interval; VAC, ventilator-associated conditions; APACHE, acute physiology and chronic health evaluation.

### VAC and Associated Mortality

**Fig 2:**
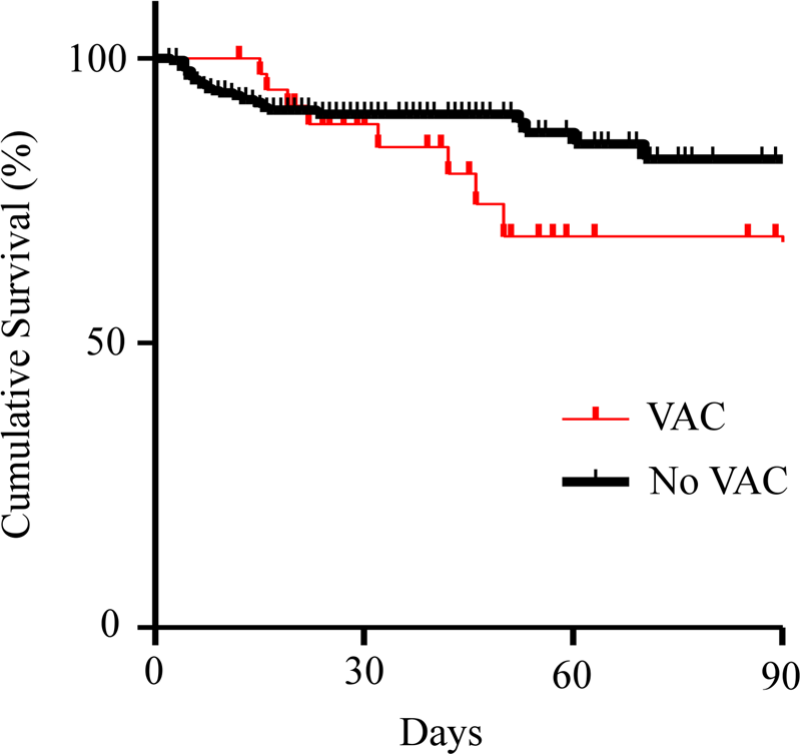
Kaplan–Meier analysis of cumulative survival.

Fig 2 depicts survival curves estimated using the Kaplan–Meier method. The survival rate rapidly declined in a linear manner (by ~50 days) in patients with VACs, whereas the rate in patients without VACs gradually declined. Between-group differences (particularly prominent after hyperacute phase) in the cumulative survival rate existed, and statistically significant differences were found using log-rank analysis (*p* = 0.036). Another test using Cox’s proportional hazard analysis with a time-dependent covariate confirmed a statistically significant difference in mortality of patients with VACs compared with those without VACs (hazard ratio = 2.623; 95% CI = 1.294–5.317; *p* = 0.0075).

## Discussion

With the intention to set a basic guideline for VAC preventive measures, a risk factor analysis was conducted. The following four risk factors were identified on multivariate analysis: (1) patient non-exposure to intensivists; (2) relatively higher mean ΔP level; (3) development of edema; and (4) a larger BW increase. Moreover, the presence or absence of VACs was related to mortality.

The etiologies of VAC in this study were pneumonia, atelectasis, ARDS, and pleural effusion. The four care-related risk factors for VAC identified in this study corresponded to well-known risk factors for each of these etiologies as members of VACs. Volume overload is associated with ARDS [14, 15], VAP [16], atelectasis [17], and pleural effusion. In this analysis, the development of edema and BW increase are extracted as a surrogate of volume overload, and this result is compatible with those reported in several previous reports [3, 8, 18]. In this study, the BW increase rate for patients with VACs was approximately 5%. According to Chittawatanarat et al., who analyzed the relationship between volume overload and respiratory complications in the ICU, the cut-off value of the BW increase rate was 5% [19]; our results concur with their finding. ARDS is developed and/or aggravated by ventilator-induced lung injury (VILI) that accompanies inappropriate MV [20, 21]. Therefore, extracting of ΔP, a risk factor for VILI, is reasonable. Lewis S et al. reported that the mode of the mandatory MV was a risk factor for VAC. The authors provided further discussion on increasing the risk for VAC because of the mandatory mode-induced VILI. Moreover, the mandatory mode generally has a larger ΔP than the modes with spontaneous breathing. Thus, identification of ΔP in our study appears to be consistent with their results. Apart from the development of VILI and ARDS, the potential for spreading and extending the pneumonia was not eliminated because lager ΔP provides the dispersion and propagation of inflammatory infiltrates and secretion via airway network in the lung [22, 23]. Participation of intensivists in the treating of ICU patients is useful for preventing VAP [24]. In addition, as intensivists have great knowledge and skills for MV, i.e., they can apply ΔP, PEEP, and associated adjacent therapies (fluid management, sedation management, etc.) most appropriately, we supposed their involvement was advantageous for preventing any other various ventilator-associated respiratory complications (i.e., VACs). Therefore, it seems that this shows why the presence or absence of or collaboration with intensivists when treating patients has a major effect on VAC. Our ICU is not a closed ICU; therefore, intensivist involvement varied from patient to patient. This result indicates that the positive involvement of intensivists in the management of MV patients can be recommended. Boyer et al., who investigated the preventability of VACs, surmised that VACs caused by volume overload, insufficient PEEP, VAP, or aspiration were preventable [8]. Moreover, Kompas et al. revealed that spontaneous awakening trials (SATs) and spontaneous breathing trials (SBTs), indicating these two could avoid ΔP and volume overload, were associated with lower prevalence of VACs [25]. In this regard, we believe that prevention of risks identified in our analysis can actually reduce VACs to some extent. We recommend that these four core factors be targeted for the prevention of VACs.

The results of the Cox proportional hazard analysis that do not involve the classification of VACs were also consistent with that of the polychotomous logistic regression analysis that involves the classification of VACs according to the incidence of early- or late-VACs, which may indicate that there were no differences for core risk factors between early- and late-VACs. Although the causes of VAP differ between early- and late-VAPs, the risk of VACs, including VAP, did not differ between early-VACs and other types of VACs. This also supports that VACs is not equivalent to VAP and encompasses a variety of respiratory complications.

Nevertheless, it was comprehensively acceptable that the external and internal validities of these results were maintained; moreover, external validity should be carefully interpreted as described further.

In this analysis, no risk factors specific to VACs were found, which could be attributable to VAC being an aggregate of distinct respiratory complications that include not only pneumonia but also various respiratory complications. Therefore, there is a potential for variation in the risks of VACs as statistically and preferentially identified, depending on the etiological components of clinical VACs. In this analysis, the major etiologies of VACs, except for pneumonia, were atelectasis (21.6%) and ARDS (13.5%). In a report by Hayashi et al., major conditions other than pneumonia were atelectasis (16.3%) and pulmonary edema (11.8%), and a care-related risk factor for VACs was fluid overload [3]. Boyer et al. reported ARDS (16.4%) and pulmonary edema (14.9%) as major conditions, and care-related risks for VACs were insufficient PEEP, fluid overload, and aspiration [8]. According to a report by Muscendere et al, wherein pneumonia accounted for a relatively large proportion (28%) of VAC, VAP-related factors such as frequency of change of humidifiers, heat moisture exchangers, and suction systems were risks factors for VACs [6]. As stated earlier, it seems safe to conclude that the four factors derived from this study are likely core factors for VAC risk. Moreover, a potential for minor variation remains in the statistically identified risks depending on the etiology of VACs. Moreover, it indicated that the VAC risk prevention initiative requires minor individual adjustments based on the details of the components implicated in VAC etiology. When addressing a quality improvement initiative for the prevention of VACs (called the VAC bundle), directly applying the traditional VAP bundle [26–30] may not be appropriate. Therefore, we believe that risk analysis should be undertaken when a VAC is monitored for an appropriate period in each ICU.

In the results of our study, factors other than host-related factors were mainly identified as risk factors for VACs; however, in general, the severity of the underlying disease is also considered to be contributory. In addition, Boyer et al., who researched the preventability of VACs, concluded that preventable VACs were only 37% of the overall VACs in their study [8]. With these taken into consideration, it is not known by how much the survival rate will be improved when the risks identified are eliminated, with a subsequent reduction in VACs achieved.

There are some limitations in this study. Although many potentially interesting variables for incidences of VACs were selected, of the 556 cases which met the inclusion criteria of CDC VAC surveillance, 253 cases were excluded because the data of the variables in the areas of interest for this analysis were not completely fulfilled. In this study, the data were automatically extracted from an electronic database. The electronic database was assembled by medical staff, such as doctors, nurses, and other medical personnel who manually enter patient records into the computer each time the care is provided via treatment and assessment at the bedside. Therefore, there could be input omission. In particular, entry tasks for care-related factors include manual entry of every care, daily and hourly from hospitalization till discharge. This requires a heavy workload, and the medical staff must do this entry work while providing care to patients, making it difficult to eliminate errors completely. Unfortunately all the variables (shown in Table 3) of all of the patients were not entered completely at all time points. Thus, the number of patients that were analyzed decreased. To improve this situation, we considered reducing the number of input explanatory variables. However, we decided against this considering that the purpose of this study was to comprehensively explore the VAC risks. There are limitations in our research methodology, such as a single center study, small group size, and 40% of the patients were excluded as a result of missing data. These limitations have the potential for a selection bias in the explorative factor analysis. This potential for bias cannot be completely ruled out in this study. Furthermore, we do not claim that five factors can fully explain complete VAC development, but there could be additional risk factors that were not identified. If we had access to a larger number of cases, we could include more factors in the analysis. In addition, if we increased the number of cases, more variables may be selected as significant factors by the stepwise procedure. We did not plan for an analysis of the optimum cut-off values for risks (i.e., the level of ΔP, the volume of infusion), and these were not covered in this study. Further investigation of the risk factors of infected VAC (iVAC) and possible or probable pneumonia VAC (pVAP), as subtypes of VAC provided by CDC guidelines (iVAC is VAC with general evidence of infection and pVAP is iVAC with lab evidence of pneumonia), was not performed due to the small number of patients; however, this should be considered as a future avenue of research.

## Conclusion

In this study, care-related factors were identified as risk factors for VAC. The results of this analysis demonstrate that key components of the VAC prevention program are the involvement of intensivists in treatment, avoidance of volume overload, and management by lower ΔP. It seems safe to conclude that these are likely core factors; however, it was suggested that minor adjustments should be made for preventive measures by each ICU because the etiology and components of VAC can vary, indicating that the care-related risk factors can vary a little by surveillance in each ICU. For the quality improvement initiative, it is crucial to verify how much the risk prevention program (VAC bundle), derived from VAC surveillance, contributes to the improvement of patient outcomes, and hence, further studies are needed.
